# Biological Predictors of Clozapine Response: A Systematic Review

**DOI:** 10.3389/fpsyt.2018.00327

**Published:** 2018-07-26

**Authors:** Ruta Samanaite, Amy Gillespie, Kyra-Verena Sendt, Grant McQueen, James H. MacCabe, Alice Egerton

**Affiliations:** ^1^Psychosis Studies Department, Institute of Psychiatry, Psychology and Neuroscience, King's College London, London, United Kingdom; ^2^Department of Psychiatry, University of Oxford, Oxford, United Kingdom

**Keywords:** clozapine, treatment response, schizophrenia, treatment-resistance, response biomarker

## Abstract

**Background:** Clozapine is the recommended antipsychotic for treatment-resistant schizophrenia (TRS) but there is significant variability between patients in the degree to which clozapine will improve symptoms. The biological basis of this variability is unknown. Although clozapine has efficacy in TRS, it can elicit adverse effects and initiation is often delayed. Identification of predictive biomarkers of clozapine response may aid initiation of clozapine treatment, as well as understanding of its mechanism of action. In this article we systematically review prospective or genetic studies of biological predictors of response to clozapine.

**Methods:** We searched the PubMed database until 20th January 2018 for studies investigating “clozapine” AND (“response” OR “outcome”) AND “schizophrenia.” Inclusion required that studies examined a biological variable in relation to symptomatic response to clozapine. For all studies except genetic-studies, inclusion required that biological variables were measured before clozapine initiation.

**Results:** Ninety-eight studies met the eligibility criteria and were included in the review, including neuroimaging, blood-based, cerebrospinal fluid (CSF)-based, and genetic predictors. The majority (70) are genetic studies, collectively investigating 379 different gene variants, however only three genetic variants (DRD3 Ser9Gly, HTR2A His452Tyr, and C825T GNB3) have independently replicated significant findings. Of the non-genetic variables, the most consistent predictors of a good response to clozapine are higher prefrontal cortical structural integrity and activity, and a lower ratio of the dopamine and serotonin metabolites, homovanillic acid (HVA): 5-hydroxyindoleacetic acid (5-HIAA) in CSF.

**Conclusions:** Recommendations include that future studies should ensure adequate clozapine trial length and clozapine plasma concentrations, and may include multivariate models to increase predictive accuracy.

## Introduction

Approximately one third of patients with schizophrenia do not respond to standard antipsychotic treatment and are classified as having treatment resistant schizophrenia (TRS) (1). Clozapine has efficacy in reducing symptoms in patients who have not responded to other antipsychotics (2–4), but carries risk of serious side effects and requires regular blood monitoring. Unfortunately, clozapine will still fail to improve symptoms in 40 to 70% of TRS patients (2, 5), and currently this can only be determined through a trial of clozapine treatment. For these reasons patients and clinicians are often reluctant to initiate clozapine treatment. For example, a recent study found that there was a delay of around 4 years between patients meeting TRS criteria and the initiation of clozapine, and that during this period patients were often treated with alternative drug regimens that are not evidence-based and are associated with adverse effects, such as antipsychotics at doses higher than the maximum recommended, and antipsychotic polypharmacy (6). If tests could be developed to help clinicians predict in advance whether or not a given patient is likely to respond to clozapine, this could substantially reduce the delay before clozapine initiation, and clozapine could be selectively employed in the subset of patients in whom it is likely to be effective.

Of course, clozapine response first requires adequate dosing; patients who have clozapine plasma concentrations of 350 ng/mL or above are more likely to show improvements in symptoms, with reported sensitivity and specificity of 64–86 and 55–78% (7–10). Nonetheless a significant proportion of patients do not improve despite having adequate clozapine plasma concentrations (9), which may be termed “clozapine resistant schizophrenia” (11). An emerging number of cross-sectional studies that have compared treatment-resistant to treatment responsive schizophrenia report biological differences at group level, which may suggest that TRS is a categorically distinct illness subtype (12), and it is possible that clozapine-resistant schizophrenia may reflect a further biological subtype. Overall, this suggests that individual biological variability may play an important role in determining the degree of clozapine response in the context of adequate dosing. This raises the possibility that biological markers may be able to predict the likelihood that symptoms will improve with clozapine treatment in advance of clozapine initiation.

Numerous studies have investigated biological predictors of response to non-clozapine antipsychotics, including symptomatic response to initial antipsychotic administration in patients with first-episode psychosis [for recent review see (13)]. The degree of antipsychotic response may be related to brain structure (14), neurochemistry (15), or activity (16–19) before starting antipsychotic treatment, or associated with genetic variability (20). However, it is unknown whether similar factors may be predictive of response to clozapine, and this is a particularly important question for clinical practice as it may encourage earlier clozapine initiation in those patients most likely to benefit, or avoidance of clozapine exposure in those unlikely to respond. Recent studies indicate that there are two distinct patterns of treatment-resistance onset, with some patients developing resistance later in their illness but the majority demonstrating resistance from illness onset (21, 22), further supporting the need to promptly identify these patients and establish their likelihood of responding to clozapine.

The purpose of this article is to provide a systematic review of studies that have investigated biological predictors of response to clozapine, in order to provide an update on the research in the area and identify the most promising areas for further investigation. We limit our scope to biological variables as predictors of response. Demographic and clinical factors may also be important in understanding some aspects of clozapine response, and these have been comprehensively reviewed elsewhere (23, 24).

## Methods

### Search strategy

The search was performed in the PubMed database on 20th January 2018 using the keywords “clozapine” AND (“response” OR “outcome”) AND “schizophrenia.” The search was limited to the titles and abstracts of the papers, with additional filters set to human studies and English language.

Abstracts were reviewed against study inclusion and exclusion criteria (below), and independently reviewed; there was an inter-rater reliability kappa of 0.914. The full text of the remaining potentially eligible studies were reviewed independently by authors RS and AG; there was 100% agreement on inclusion of the final studies. Reference lists were hand-searched to identify additional studies.

### Study selection

Inclusion required that studies were published in English in peer-reviewed academic journals. Inclusion also required that studies examined a biological variable in relation to clozapine response. Only studies that measured clozapine response as a change in positive, negative or overall symptom severity or global functioning were included. For biological variables such as brain activity or metabolite concentrations in blood, which may be affected by clozapine treatment, inclusion required that these measures were acquired prospectively, before clozapine initiation. For genetic variables, cross-sectional studies of clozapine response were also included. Studies were included if they investigated either clozapine monotherapy or clozapine in combination with other pharmacological or non-pharmacological interventions, as is reflective of clinical practice.

Data reported only in editorials, review articles, conference abstracts, conference reports, news articles, meta-analyses, or other non-primary data formats were excluded. Where more than one article reported data in overlapping patient samples, only the study with the largest sample was included. Studies were also excluded if the samples included a combination of patients taking only non-clozapine antipsychotics and clozapine-treated patients, without reporting results for clozapine-treated patients separately.

### Data extraction

Data were extracted into an Excel database. The following data were extracted: the biological predictor variable(s), sample size, availability of plasma clozapine concentrations (yes/no), mean plasma clozapine concentrations, mean clozapine dose, duration of clozapine treatment (months), the clozapine response criteria used, and whether results were statistically significant.

For review, articles were categorized into neuroimaging, blood-based, cerebrospinal fluid-based, cardiac, and genetic markers.

## Results

**Figure 1:**
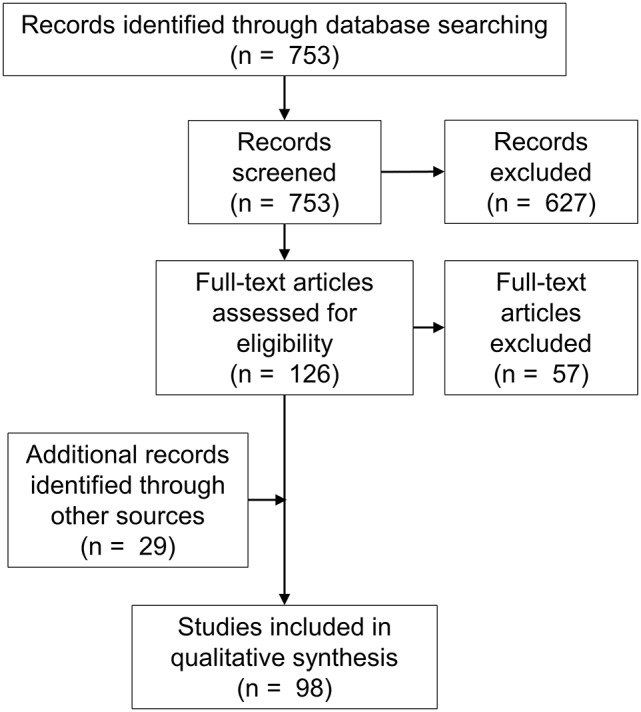
PRISMA diagram.

The search returned 753 articles. Abstract review identified 126 potentially eligible studies, and subsequent full-text screening identified 69 eligible studies. The excluded studies are listed in Table 1. Twenty-nine additional eligible articles were identified via other means including hand-searches of reference lists (Figure 1).

**Table 1:** Excluded studies.

**First Author, Year**	**Title**	**Exclusion reason**
(25)	Progressive Brain Atrophy and Cortical Thinning in Schizophrenia after Commencing Clozapine Treatment.	Compares longitudinal changes after initiation not baseline variation
Ajami, 2014	Changes in serum levels of brain derived neurotrophic factor and nerve growth factor-beta in schizophrenic patients before and after treatment.	Results include non-clozapine medication
Blessing, 2011	Atypical antipsychotics cause an acute increase in cutaneous hand blood flow in patients with schizophrenia and schizoaffective disorder.	On clozapine at baseline
Buchsbaum, 1992	Effects of clozapine and thiothixene on glucose metabolic rate in schizophrenia.	Cannot obtain full-text to confirm
Curtis, 1995	Effect of clozapine on d-fenfluramine-evoked neuroendocrine responses in schizophrenia and its relationship to clinical improvement.	Compares longitudinal changes after initiation not baseline variation
Delieu, 2001	Antipsychotic drugs result in the formation of immature neutrophil leucocytes in schizophrenic patients.	Does not measure outcome
Dursun, 1999	The effects of clozapine on levels of total cholesterol and related lipids in serum of patients with schizophrenia: a prospective study.	Does not report results for response
(26)	The effect of clozapine on neuroimaging findings in schizophrenia.	Cannot obtain full-text to confirm
Frieboes, 1999	Characterization of the sigma ligand panamesine, a potential antipsychotic, by immune response in patients with schizophrenia and by sleep-EEG changes in normal controls.	Does not investigate clozapine
(27)	Prefrontal sulcal prominence is inversely related to response to clozapine in schizophrenia.	Does not specify when biological variable measured
Ghaleiha, 2011	Correlation of adenosinergic activity with superior efficacy of clozapine for treatment of chronic schizophrenia: a double blind randomized trial.	Compares biological variable after initiation
Gothelf, 1999	Clinical characteristics of schizophrenia associated with velo-cardio-facial syndrome.	No variation in biological variable
Gothert, 1998	Genetic variation in human 5-HT receptors: potential pathogenetic and pharmacological role.	Not primary research - review
Graff-Guerrero, 2009	The effect of antipsychotics on the high-affinity state of D2 and D3 receptors: a positron emission tomography study With [11C]-(+)-PHNO.	Cross-sectional
Gross, 2004	Clozapine-induced QEEG changes correlate with clinical response in schizophrenic patients: a prospective, longitudinal study.	Compares longitudinal changes after initiation not baseline variation
(28)	Regional cortical anatomy and clozapine response in refractory schizophrenia.	Does not specify when biological variable measured
Hsu, 2000	No evidence for association of alpha 1a adrenoceptor gene polymorphism and clozapine-induced urinary incontinence.	Outcome not therapeutic response
Jacobsen, 1997	Cerebrospinal fluid monoamine metabolites in childhood-onset schizophrenia.	Compares longitudinal changes after initiation not baseline variation
Jenkins, 2014	Identification of candidate single-nucleotide polymorphisms in NRXN1 related to antipsychotic treatment response in patients with schizophrenia.	Does not investigate clozapine
Jones, 1998	Neuroendocrine evidence that clozapine's serotonergic antagonism is relevant to its efficacy in treating hallucinations and other positive schizophrenic symptoms.	Does not specify when biological variable measured
Joober, 1999	T102C polymorphism in the 5HT2A gene and schizophrenia: relation to phenotype and drug response variability.	Does not investigate clozapine
Knott, 2001	Quantitative EEG in schizophrenia and in response to acute and chronic clozapine treatment.	Does not report results for response
Knott, 2002	EEG coherence following acute and chronic clozapine in treatment-resistant schizophrenics.	Overlapping sample with other study
Lahdelma, 1998	Association between HLA-A1 allele and schizophrenia gene(s) in patients refractory to conventional neuroleptics but responsive to clozapine medication.	Does not measure outcome
Lahdelma, 2001	Mitchell B. Balter Award. Human leukocyte antigen-A1 predicts a good therapeutic response to clozapine with a low risk of agranulocytosis in patients with schizophrenia.	No clozapine non-responders
(29)	Clozapine but not haloperidol Re-establishes normal task-activated rCBF patterns in schizophrenia within the anterior cingulate cortex.	Does not report results for response
Lally, 2013	Increases in triglyceride levels are associated with clinical response to clozapine treatment.	Compares longitudinal changes after initiation not baseline variation
Lauriello, 1998	Association between regional brain volumes and clozapine response in schizophrenia.	Compares biological variable after initiation
Machielsen, 2014	The effect of clozapine and risperidone on attentional bias in patients with schizophrenia and a cannabis use disorder: An fMRI study.	Does not report results for response
Maes, 1997	*In vivo* immunomodulatory effects of clozapine in schizophrenia.	Does not specify when biological variable measured
Maes, 2002	Increased serum interleukin-8 and interleukin-10 in schizophrenic patients resistant to treatment with neuroleptics and the stimulatory effects of clozapine on serum leukemia inhibitory factor receptor.	Does not specify when biological variable measured
Malow, 1994	Spectrum of EEG abnormalities during clozapine treatment.	Does not measure outcome
Markianos, 1999	Switch from neuroleptics to clozapine does not influence pituitary-gonadal axis hormone levels in male schizophrenic patients.	Compares longitudinal changes after initiation not baseline variation
Meltzer, 1993	The cimetidine-induced increase in prolactin secretion in schizophrenia: effect of clozapine.	Does not measure outcome
Molina, 2008	Clozapine may partially compensate for task-related brain perfusion abnormalities in risperidone-resistant schizophrenia patients.	Compares longitudinal changes after initiation not baseline variation
Monteleone, 2004	Long-term treatment with clozapine does not affect morning circulating levels of allopregnanolone and THDOC in patients with schizophrenia: a preliminary study.	Does not report results for response
Mouaffak, 2011	Association of an UCP4 (SLC25A27) haplotype with ultra-resistant schizophrenia.	Results include non-clozapine medication
(30)	The SNAP-25 gene may be associated with clinical response and weight gain in antipsychotic treatment of schizophrenia.	Results include non-clozapine medication
Murad, 2001	A family-based study of the Cys23Ser 5HT2C serotonin receptor polymorphism in schizophrenia.	Does not measure outcome
Niznikiewicz, 2005	Clozapine action on auditory P3 response in schizophrenia.	Does not measure outcome
Ozdemir, 2001	Treatment-resistance to clozapine in association with ultrarapid CYP1A2 activity and the C–>A polymorphism in intron 1 of the CYP1A2 gene: effect of grapefruit juice and low-dose fluvoxamine.	Individual case report
Patel, 1997	Chronic schizophrenia: response to clozapine, risperidone, and paroxetine.	Individual case report
Paunovia, 1991	Neuroleptic actions on the thyroid axis: different effects of clozapine and haloperidol.	Does not measure outcome
Pedrini, 2011	Serum brain-derived neurotrophic factor and clozapine daily dose in patients with schizophrenia: a positive correlation.	Does not report results for response
Peet, 2002	A dose-ranging exploratory study of the effects of ethyl-eicosapentaenoate in patients with persistent schizophrenic symptoms.	Does not report results for response
Pickar, 1994	Clinical response to clozapine in patients with schizophrenia.	Does not investigate clozapine
Pilowsky, 1992	Clozapine, single photon emission tomography, and the D2 dopamine receptor blockade hypothesis of schizophrenia.	Does not report results for response
Procyshyn, 2007	Changes in serum lipids, independent of weight, are associated with changes in symptoms during long-term clozapine treatment.	Results include non-clozapine medication
Reynolds, 1996	The importance of dopamine D4 receptors in the action and development of antipsychotic agents.	Not primary research - review
Risby, 1995	Clozapine-induced EEG abnormalities and clinical response to clozapine.	No variation in biological variable
Ruderfer, 2016	Polygenic overlap between schizophrenia risk and antipsychotic response: a genomic medicine approach.	Does not measure outcome
Schulz, 1997	Blood biogenic amines during clozapine treatment of early-onset schizophrenia.	Overlapping sample with other study
Sun 2016	Diurnal neurobiological alterations after exposure to clozapine in first-episode schizophrenia patients.	Does not report results for response
Swerdlow, 2006	Antipsychotic effects on prepulse inhibition in normal 'low gating' humans and rats.	Does not investigate clozapine
Szekeres, 2004	Role of dopamine D3 receptor (DRD3) and dopamine transporter (DAT) polymorphism in cognitive dysfunctions and therapeutic response to atypical antipsychotics in patients with schizophrenia.	Results include non-clozapine medication
Treves, 1996	EEG abnormalities in clozapine-treated schizophrenic patients.	Compares biological variable after initiation
Zahn, 1993	Autonomic effects of clozapine in schizophrenia: comparison with placebo and fluphenazine.	Does not specify when biological variable measured

### Study characteristics

Ninety-eight studies met the inclusion criteria, for which the methodological details are provided in Tables 2, 4, 6, and 8. Of these, 70 studies investigated genetic variables, 16 studies investigated blood or CSF-based variables, 11 studies investigated neuroimaging markers, and 1 investigated a cardiac variable. Sample sizes ranged from 7 (42) to 591 participants (43). Studies included participants from across Europe (Britain, Turkey, Italy, Spain, Germany), America, Canada, and Asia (China, Israel, India, Taiwan, Pakistan).

**Table 2:** Included neuroimaging studies.

**Study**	**Imaging variables**	**Participant sample**	**Minimum clozapine trial**	**Outcome measure**	**Clozapine dose**	**Plasma clozapine**
(31)	MRI (caudate, prefrontal cortex, hippocampal volume)	17 White American 5 African American	10 weeks	BPRS, SANS	200–600 mg	Not reported
(32)	SPECT and MRS (frontal, parietal, temporal, and occipital lobes, the caudate, thalami, and cerebellum)	22 Turkish	8 weeks	PANSS	390.48 mg (mean)	Not reported
(33)	EEG	10 Korean	4 weeks	BPRS 20% reduction	Responders: 265.6 mg (mean) Non-responders: 204.2 (mean)	Not reported
(34)	EEG	37 Canadian	Unspecified	Absolute score on PANSS (varied with machine learning model), quantitative clinical assessment score 25% reduction	50–600 mg	Not reported
(35)	EEG	13 Canadian	6 weeks	PANSS	381.25 mg (mean)	Not reported
(36)	CT (prefrontal and general sulci widening)	36 American	6 months	CGI- Change ≥ 2	491 mg (mean)	Not reported
(37)	PET (dorsolateral prefrontal, temporal, hippocampal, thalamus, caudate and pallidum/putamen regions) MRI (dorsolateral prefrontal temporal, and hippocampal regions)	25 Spanish	6 months	SAPS and SANS	250–600 mg	Not reported
(38)	MRI (frontal—superior, caudal middle, rostral middle, pars opercularis, pars triangularis, pars orbitalis, lateral orbital, medial orbital; temporal—superior temporal, entorhinal, parahippocampal; cingulate—caudal anterior, rostral anterior; and occipital—lateral occipital and lingual)	11 European	1 year	PANSS	220.45 mg (mean)	Not reported
(39)	EEG	86 American	Unspecified	GAF	Not reported	Not reported
(40)	EEG	47 Canadian	1 year	PANSS 35% reduction	347 mg (mean)	Not reported
(41)	SPECT (orbitofrontal, superior dorsolateral prefrontal, anterior prefrontal, inferior dorsolateral prefrontal, thalamic, and basal ganglia regions)	39 Spanish	26 weeks	SAPS and SANS 50% reduction + CGI <3	551 mg (mean)	Not reported

*BPRS, Brief Psychiatric Rating Scale; CGI, Clinical Global Impression; CT, Computerized Tomography; EEG, Electroencephalography; GAF, Global Assessment of Functioning; MRI, Magnetic Resonance Imaging; MRS, Magnetic Resonance Spectroscopy; PANSS, Positive and Negative Syndrome Scale; PET, Positron Emission Tomography; SAPS, Scale for the Assessment of Positive Symptoms; SANS, Scale for the Assessment of Negative Symptoms; SPECT, Single-Photon Emission Computed Tomography*.

As detailed in Tables 2, 4, 6, and 8, clozapine trial length varied from 4 weeks (44) to 16 months (45). Only nine studies (9%) reported clozapine plasma levels; of these, six gave a group mean (46–51) and three reported the mean dose for a responder and non-responder group separately (52–54). Sixty-three studies (64%) reported data on clozapine doses. Of these, 21 reported the dose range across the sample (e.g., 150–600 mg) while 36 reported the group mean and 6 reported the mean dose for a responder and non-responder group separately.

The primary outcome variables for determining clozapine response varied considerably (Tables 2, 4, 6, and 8). Thirteen studies used a combination of outcome measures to define clozapine response, and one used different outcome measures for different participants (55).

### Neuroimaging predictors of clozapine response

Eleven neuroimaging studies met the inclusion criteria (Tables 2, 3). These included four structural imaging studies, three single photon emission computerized tomography or positron emission tomography (SPECT/PET) studies of brain perfusion or metabolism, one proton magnetic resonance spectroscopy (1H-MRS) study of brain metabolite concentrations, and five electro-encephalography (EEG) studies. The length of clozapine treatment in the neuroimaging studies ranged from 4 weeks (33) to 1 year (37, 38), but none reported plasma clozapine levels.

**Table 3:** Results from neuroimaging studies.

**Imaging modality**	**Brain area**	**Studies**	**Significant Findings**	**Association with good response**
MRI	Prefrontal Cortex	(31)	Y	Greater right gray matter
		(37)	Y	Greater volume (dorsolateral)
	Frontal (superior, caudal middle, rostral middle, pars opercularis, pars triangularis, pars orbitalis, lateral orbital, medial orbital)—cortical thickness	(38)	Y	Thinner cortical thickness (right pars orbitalis)
	Hippocampus	(31)	N	–
		(37)	Y	Lower volume
	Temporal lobe (gray)	(37)	Y	Greater gray matter volume
	Temporal (superior temporal, entorhinal, parahippocampal)—cortical thickness	(38)	N	–
	Caudate	(31)	N	–
	Cingulate (caudal anterior, rostral anterior)	(38)	N	–
	Occipital (lateral occipital and lingual)	(38)	N	–
	Total intracranial volume	(37)	Y	Lower ICV
PET	Hippocampus	(37)	N	–
	Thalamus	(37)	N	–
	Pallidum/putamen	(37)	N	–
	Caudate head	(37)	N	–
	Dorsolateral prefrontal	(37)	Y	Greater activity
	Temporal	(37)	N	–
CT	General sulci widening	(36)	N	–
	Prefrontal sulci widening	(36)	Y	Lower widening
SPECT	Orbitofrontal	(41)	N	–
	Frontal	(32)	Y	Higher perfusion
	Parietal	(32)	N	–
	Temporal	(32)	N	–
	Occipital	(32)	N	–
	Caudate	(32)	N	–
	Cerebellum	(32)	N	–
	Superior dorsolateral prefrontal	(41)	Y	Higher right perfusion
	Anterior prefrontal	(41)	N	–
	Inferior dorsolateral prefrontal	(41)	Y	Higher left perfusion
	Basal ganglia	(41)	Y	Higher perfusion
	Thalamus	(41)	Y	Higher perfusion
		(32)	Y	Higher perfusion
EEG	Unspecified	(39)	Y	Abnormal EEG, better response
	Correlation dimensions, primary lyapunov exponent, and mutual cross prediction with electrodes at Fpl, Fp2, C3, C4, O1, and O2	(33)	No statistical analysis	Non-frontal-driving and occipital response patterns associated with better response (significance testing not done)
	Machine learning approach with electrodes at Fp1, Fp2, F3, F4, F7, F8, T3, T4, C3, C4, T5, T6, P3, P4, O1, and O2	(34)	Y	Discriminating variables: mutual information between T3 & P3, T3 & O1, C3 & P3, F8 & T4; coherence between T3 & O1, T3 & P3, C3 & O1, F3 & P3, T6 & P3, T3 & O1, T3 & T5, C3 & P3, F7 & F3; and left to right PSD-ratio, T5/T6
	Intra and inter hemispheric asymmetry with electrodes at F3, F4, F7, F8, T3, T4, C3, C4, T5, T6, P3, P4, O1, and O2	(35)	Y	Greater interhemispheric central anterior temporal theta and beta ratios, better response. Greater intra-hemispheric frontal-anterior temporal and anterior temporal mid temporal delta ratios, and across majority of regions theta ratios, better response.
	Machine learning approach with electrodes at Fp1, Fp2, F7, F3, Fz, F4, F8, T7, C3, Cz, C4, T8, P7, P3, Pz, P4, P8, O1, O2	(40)	Y	Increased joint activity between midline fronto-polar and anterior temporal right, midline fronto-polar and parietal right, midline fronto-polar and frontal midline, central midline and parietal right, midline occipital-polar and parietal right

*CT, Computerized Tomography; EEG, Electroencephalography; MRI, Magnetic Resonance Imaging; PET, Positron Emission Tomography; SPECT, Single-Photon Emission Computed Tomography*.

#### Brain structure

The first published study used computerized tomography (CT) to examine sulcal widening as a predictor of clozapine response (36). A good clozapine response was associated with significantly lower widening scores in the prefrontal sulci compared to a poor response, suggesting that poor clozapine response may be associated with a higher degree of frontal atrophy. Three more recent studies used structural magnetic resonance imaging (MRI) to predict clozapine response. In a clinical trial of clozapine vs. haloperidol, Arango et al. (31) found that larger right prefrontal cortical gray matter volumes were associated with greater reduction in SANS total scores after treatment in the clozapine group. No associations were found with positive symptoms, or for relationships between symptoms and caudate, hippocampal or total intracranial volumes. Molina et al. (37) investigated associations between regional brain volume and clozapine response. Temporal cortex volume was directly associated with improvement in positive symptoms, whereas dorsolateral prefrontal cortical (DLPFC) cerebrospinal fluid (CSF) content was inversely associated with improvement in positive symptoms. DLPFC volume was directly associated with improvement in negative symptom severity, and the intracranial volume was negatively related to improvement in disorganization syndrome.

These studies therefore provide a generally consistent picture that greater volumes, particularly in frontal cortical regions, are associated with a better response to clozapine treatment. However, Molina et al. (38) found that thinner baseline right pars orbitalis cortex predicted greater improvement in PANSS scores following at least 1 year clozapine use in antipsychotic-naïve first-episode patients. This difference might be explained by the different patient populations, with the two former studies including treatment-resistant patients with previous antipsychotic exposure and the latter including antipsychotic-naïve patients who may have responded to conventional antipsychotics.

#### Brain perfusion and metabolism

Regional brain perfusion and metabolism were also investigated as predictors of clozapine response. Rodriguez et al. (41), in an extension of an earlier report (56), used 99mTc-HMPAO single photon emission computed tomography (SPECT) to measure regional brain perfusion as a predictor of response to clozapine. Compared to the non-responder group, responders had higher baseline perfusion in right lower DLPFC, left upper DLPFC, thalamus, and left and right basal ganglia. Discriminant analysis showed that perfusion in the thalamus and right DLPFC distinguished between responders and non-responders with 78.9% accuracy. Similarly, Ertugrul et al. (32), also employing Tc-99m HMPAO SPECT imaging, reported that increased levels of perfusion in the right frontal cortex and thalamus were associated with greater improvement in PANSS score with clozapine treatment. Molina et al. (37), using 18F-deoxyglucose (18F-DG) positron emission tomography (PET), found that baseline metabolic rate in the DLPFC was directly related to improvement in negative symptoms, however no associations were found between metabolism in other brain regions, or with improvement in positive or disorganization symptoms. This finding of a direct association between DLPFC metabolic rate and clozapine response is consistent with findings of a direct association between DLPFC perfusion and clozapine response (32, 41).

#### Magnetic resonance spectroscopy

One 1H-MRS study investigated whether metabolite concentrations in the DLPFC may predict response to clozapine (32). In this sample of 22 patients, neither the concentration of n-acetyl aspartate (NAA) nor choline was predictive of the subsequent degree of change in symptoms on the PANSS. Relationships with other metabolites in the 1H-MRS spectrum, including glutamate, were not reported.

#### Electroencephalography

Five EEG studies, investigating a range of variables related to brain electrical activity, including EEG abnormalities and hemispheric asymmetry, were included (33–35, 39, 40). The first EEG study (39) investigated whether clozapine response was predicted by the presence of minor EEG abnormalities, defined as focal or generalized slowing or sharp waves, focal dysrhythmias, spikes, and spike-wave patterns. There were no overall differences in clozapine response between patients with normal compared to abnormal EEG, however secondary analysis found that in female participants, improvements in GAF score were greater in those with a normal EEG before clozapine treatment. Knott et al. (35) reported that improvements in PANSS positive, negative symptoms and global psychopathology were related to greater intrahemispheric frequency asymmetries. Kang et al. (33) ran mutual cross-prediction analysis to identify if activity in one channel was driving the dynamics of another channel. The sample was too small to conduct significant testing, but they observed that the group of participants without a frontal-driving system and occipital response system had a higher proportion of responders to clozapine. A fourth EEG study of clozapine response (34) applied a machine-learning algorithm to distinguish clozapine responders and non-responders based on their pre-treatment EEG measures, using first the leave-one-out cross-validation procedure and then two independent datasets to train and test the classifiers. This algorithm successfully distinguished these groups with more than 85% accuracy. The authors reported a list of 20 EEG measures that were found to have the greatest predictive value, which mainly included measures of the left temporal areas. Similarly, Ravan et al. (40) applied a machine-learning algorithm to patients' EEG data from before and after a year of clozapine treatment. The most-responsive patients had five “discriminating features” at baseline; these were predominantly in the beta-band, with the most dominant features joint activity between the pre-frontal and right parietal or right anterior temporal regions.

### CSF-based predictors of clozapine response

*A priori* selection of CSF- and peripheral predictive biomarkers of clozapine response has been driven by clozapine's “atypical” pharmacological profile of high affinity at serotonin 5-HT_2A_ receptors in combination with lower affinity at dopamine D_2_ receptors (57). Our search returned three studies of CSF biochemicals in predicting clozapine response (Tables 4, 5). Two of these studies provided data on plasma clozapine concentrations (47, 48). Sample sizes in these studies ranged from 10 (64) to 21 participants (47), and all used the BPRS to measure symptomatic improvement.

**Table 4:** Included blood or CSF-based studies.

**Study**	**Blood or CSF based variables**	**Participant sample**	**Minimum clozapine trial**	**Outcome measure**	**Clozapine dose**	**Plasma clozapine**
(58)	Platelet 5-HT2 receptor binding (plasma)	11 American	6 weeks	BPRS	Not reported	Not reported
(59)	HVA, MHPG, noradrenaline, cortisol, prolactin (plasma)	14 American	6 weeks	PANSS	300-900 mg	Not reported
(26)	Serotonin (plasma, platelet, MAO)	20 Turkish	8 weeks	PANSS, CGI	382.5 mg (mean)	Not reported
(42)	Aspartate, glutamate and glycine (serum)	7 American	8 months (mean)	BPRS, SANS	393 mg (mean)	Not reported
(52)	Adrenaline, noradrenaline, dopamine, MHPG (plasma) Serotonin (serum)	15 German adolescents	6 weeks	BPRS 20% reduction and total <34	100–600 mg	Responders: 114 ng/mL (mean) Non-responders: 128 ng/mL (mean)
(60)	HVA, MHPG, dopamine and noradrenaline(plasma)	8 American	12 weeks	BPRS 20%	325–500 mg	Not reported
(61)	MCPP challenge: ACTH Prolactin (plasma)	19 American	5 weeks	CGI 1 point reduction	584.2 mg (mean)	Not reported
(46)	Leukocytes and neutrophils	20 Italian	8 weeks	BPRS, SAPS and SANS	365.mg (mean)	321.45 ng/mL
(62)	Human leukocyte antigen typing	50 Jewish Israeli	12 weeks	CGI score 1 or 2	>600 mg	Not reported
(63)	MCPP challenge: Cortisol, prolactin (plasma)	15 American	45–149 days	BPRS	440 mg (mean)	Not reported
(47)	HVA, 5-HIAA, MHPG and noradrenaline (CSF) HVA, noradrenaline (plasma) Prolactin (serum)	21 American	14 weeks	BPRS 20% reduction AND BPRS score less than 36 or Bunney-Hamburg Global Psychosis Rating of less than 6 (mild psychosis)	225–600 mg	430 ng/mL (mean)
(64)	HVA, 5-HIAA (CSF)	10 American	42 weeks	BPRS	450–650 mg	Not reported
(65)	HVA (plasma)	18 American	6 months	BPRS 20% reduction	Responders: 507.1 mg (mean) Non-responders: 468.2 mg (mean)	Not reported
(66)	Glycine, serine (plasma)	44 American	6 weeks	SANS, BPRS	353.7 mg (mean)	Not reported
(48)	HVA, 5-HIAA (CSF) HVA (plasma)	19 American	6 weeks	BPRS 20% reduction, CGI ≥ 3	404 mg (mean)	253 ng (mean at 3 weeks)
(67)	Prolactin, growth hormone (plasma)	7 White American 3 African American	12 weeks	BPRS	591.7 mg (mean)	Not reported

*5-HIAA, 5-Hydroxyindoleacetic Acid; ACTH, Adrenocorticotropic Hormone; BPRS, Brief Psychiatric Rating Scale; CGI, Clinical Global Impression; CSF, Cerebrospinal Fluid; GAF, Global Assessment of Functioning; HVA, Homovanillic Acid; MAO, Monoamine Oxidase; MCPP, Meta-Chlorophenylpiperazine; MHPG, 3-Methoxy-4-hydroxyphenylglycol; PANSS, Positive and Negative Syndrome Scale; SAPS, Scale for the Assessment of Positive Symptoms; SANS, Scale for the Assessment of Negative Symptoms*.

**Table 5:** Results from CSF and blood-based studies.

		**Studies**	**Significant Findings**	**Association with good response**
ACTH		(61)	Y	Greater increase after MCPP challenge
CSF	5-HIAA	(47)	N	–
		(64)	N	–
		(48)	N	–
	HVA	(47)	N	–
		(64)	N	–
		(48)	N	–
	HVA:5-HIAA	(47)	Y	Low ratio
		(64)	Y	Low ratio
		(48)	Y	Low ratio
	MHPG	(47)	N	–
	Noradrenaline	(47)	N	–
HLA typing		(62)	N	–
Leukocytes		(46)	N	–
Neutrophils		(46)	N	–
Plasma	Adrenaline	(52)	Y	Low concentration
		(60)	Y	Low concentration
	Cortisol	(59)	N	–
		(63)	Y	Greater increase after MCPP challenge
	Dopamine	(52)	N	–
		(60)	N	–
	Glycine	(66)	Y	Higher concentration
	Growth hormone	(67)	Y	Greater increase after apomorphine challenge
	HVA	(59)	Y	Lower concentration (neg symptoms)
		(60)	Y	Higher concentration
		(47)	Y	Lower concentration (in responders)
		(65)	Y	–
		(48)	N	–
	MHPG	(59)	N	–
		(52)	N	–
		(60)	N	–
	Noradrenaline	(59)	N	–
		(52)	N	–
		(60)	N	–
		(47)	N	–
	Prolactin	(59)	N	–
		(61)	N	–
		(63)	N	–
		(67)	Y	Greater decrease after apomorphine challenge
	Serine	(66)	N	–
	Serotonin	(26)	N	–
Platelets	MAO	(26)	Y	Higher concentration
	Serotonin	(26)	Y	Lower concentration
		(58)	Y	Lower receptor availability
Serum	Aspartate	(42)	N	–
	Glutamate	(42)	N	–
	Glycine	(42)	Y	Lower concentration
	Prolactin	(47)	N	–
	Serotonin	(52)	N	–

*5-HIAA, 5-Hydroxyindoleacetic Acid; ACTH, Adrenocorticotropic Hormone; CSF, Cerebrospinal Fluid; HVA, Homovanillic Acid; MAO, Monoamine Oxidase; MCPP, Meta-Chlorophenylpiperazine; MHPG, 3-Methoxy-4-hydroxyphenylglycol*.

#### CSF monoamines

All three studies investigated the dopamine metabolite homovanillic acid (HVA), and the serotonin metabolite 5-hydroxyindoleacetic acid (5-HIAA) (47, 48, 64). None of these studies found that HVA nor 5-HIAA concentrations alone were predictive of clozapine response. The ratio between HVA and 5-HIAA was also investigated. In all studies, lower HVA/5-HIAA concentration ratios before clozapine were associated with a greater degree of subsequent symptomatic improvement, both in the short- and longer-term (47, 48, 64). This suggests that the balance between dopamine and serotonin metabolism before clozapine administration may be predictive of clozapine response, with lower levels of dopamine metabolism relative to higher levels of serotonin metabolism being associated with better outcomes.

One study also investigated concentrations of the noradrenaline metabolite 3-methoxy-4-hydroxyphenylglycol (MHPG) in relation to clozapine response, and found no association (47).

#### CSF hormones

A single study investigated CSF prolactin concentrations as a predictor of clozapine response and found no association (47).

### Blood-based predictors of clozapine response

Our search returned 11 studies which investigated biochemicals in plasma, serum or platelets as predictors of clozapine response (Tables 4, 5). As for CSF approaches, these peripheral studies have also focussed on dopaminergic and serotonergic measures. Sample sizes in these studies ranged from 7 (42) to 50 participants (62), and all except Kahn et al. (61) and Ertugrul et al. (26) used the BPRS to measure symptomatic improvement. Data on plasma clozapine concentrations were unavailable in all but three of the studies (47, 48, 52).

#### Blood monoamines

Several studies have investigated peripheral dopaminergic variables as predictors of clozapine response, with overall negative or inconclusive findings. Two studies investigated plasma dopamine concentrations, both with negative findings (52, 60). The five studies which investigated concentrations of the dopamine metabolite HVA in plasma have reported mixed findings. Pickar et al. (47) initially reported that lower baseline plasma HVA concentrations were associated with greater reductions in symptoms, but three later studies reported that higher baseline plasma HVA concentrations were associated with greater symptom reduction (59, 60, 65), although one study found this only for negative symptoms (59) and one study found this only as a correlation with positive symptoms within the clozapine responder group (65). A further study found no association between plasma HVA and clozapine response (48). One study investigated concentrations of platelet monoamine oxidase B (MAO-B) which metabolizes dopamine (68), and found a positive association with symptom improvements following clozapine (26). Finally, as a dopaminergic pharmacological challenge, apomorphine-induced prolactin suppression and growth hormone secretion predicted better clozapine response in a preliminary study (67).

In terms of peripheral serotonergic studies, Ertugrul et al. (26) found no association with plasma serotonin concentrations and clozapine response as did an earlier study of serum serotonin concentrations in children and adolescents (52). However, Ertugrul et al. (26) also reported a negative correlation between platelet serotonin concentrations, (reflecting uptake of plasma serotonin through platelet serotonin transporters) and improvement in positive symptoms following clozapine. Arora and Meltzer (58) measured platelet 5HT_2_ receptor binding in platelet-rich plasma and reported that a lower number of 5HT_2_ binding sites before clozapine initiation was associated with poorer treatment outcomes.

Pharmacological serotonin challenge using the non-selective 5-HT receptor agonist m-chlorophenylpiperazine (mCPP) has also been employed to investigate clozapine response (61, 63). mCPP-induced adrenocorticotropic hormone (ACTH) release (61) and plasma cortisol (63) were directly associated with improvement in symptoms. In contrast, there was no association between MCPP-induced prolactin increase and clozapine response in either study (61, 63). The finding of increased MCPP-responses would suggest that elevated 5-HT system function is associated with better clinical responses to clozapine.

Finally, four studies investigated adrenaline, noradrenaline or MHPG concentrations. Two studies reported that low plasma adrenaline concentrations associate with better clozapine response (52, 60). In contrast, studies have found no association between plasma noradrenaline concentrations (47, 52, 59, 60), or plasma MHPG and clozapine response (52, 59, 60).

#### Blood glutamatergic amino acids

The glutamatergic amino acids glycine and serine act as endogenous co-agonists at the N-methyl-D-aspartate (NMDA) glutamate receptor complex, which is thought to be hypofunctional in schizophrenia and therefore increasing glycine or serine levels may have therapeutic potential (69). Two studies (42, 66) investigated glycine and serine concentrations in relation to clozapine response, from serum and plasma respectively, and have produced conflicting evidence. In a sample of 7 patients, Evins et al. (42) found that lower serum glycine concentrations predicted a better response to clozapine, whereas in the larger and longer-term study of Sumioyshi et al. (66) higher plasma glycine concentrations and higher plasma glycine/serine ratios predicted greater negative symptom improvements, whereas no associations were found between serine concentrations and clozapine response. Evins et al. (42) also measured glutamate and aspartate concentrations and report no significant associations.

#### Blood hormones

One study investigated serum prolactin levels (47) and another investigated plasma prolactin and cortisol levels (59). Neither of these studies reported significant associations with clozapine response.

#### Blood immunological variables

Two studies have looked at immological variables as predictors of clozapine response. Mauri et al. (46) measured neutrophil and leukocyte numbers before 8 weeks of clozapine treatment in 20 patients. They do not report significance testing but provide summary statistics; independent *t*-tests using this data indicates no association with response to clozapine. Meged et al. (62) investigated human leukocyte antigen (HLA) type in 50 Israeli patients but found no association between HLA type and response to clozapine after 12 weeks.

### Cardiac predictors of clozapine response

One study investigated heart rate variability in 40 participants with treatment-resistant schizophrenia using ECG (70) but did not find any pre-clozapine differences in heart rate variability associated with changes in BPRS after 8 weeks of clozapine treatment (Tables 6, 7).

**Table 6:** Included cardiac studies.

**Study**	**Cardiac variable**	**Participant sample**	**Minimum clozapine trial**	**Outcome measure**	**Clozapine dose**	**Plasma clozapine**
(70)	ECG: heart-rate variability	40 Korean	8 weeks	PANSS	Responders: 250 mg (mean) Non-responders: 266 mg (mean)	Not reported

*ECG, Electrocardiogram; PANSS, Positive and Negative Syndrome Scale*.

**Table 7:** Results from cardiac studies.

		**Studies**	**Significant findings**	**Association with good response**
ECG	Heart rate variability	55	N	–

*ECG, Electrocardiogram*.

### Genetic predictors of clozapine response

We identified a total of 70 studies investigating associations between genetic variants and clozapine response (Tables 8–11). In the first study of its kind, Frank et al. (83) recently reported that higher genetic risk of schizophrenia, calculated as the schizophrenia polygenic risk score (131), was associated with a poorer degree of response to clozapine^1^. Butcher et al. (82) recently reported that individuals with a large chromosomal deletion (22q11.2) respond as well to clozapine as patients with schizophrenia who do not have this deletion.

**Table 8:** Included genetic studies.

**Study**	**Genetic variant**	**Participant sample**	**Minimum clozapine trial**	**Outcome measure**	**Clozapine dose**	**Plasma clozapine**
(71)	HTR2A	149 White European	3 months	GAS 20-point improvement	125–600 mg	Not reported
(72)	CYP2D6	123 White European	2 months	GAS 20-point improvement	125–600 mg	Not reported
(73)	HTR2A	153 White European	Not reported	GAS 20-point improvement	125–600 mg	Not reported
(55)	DRD2	151 White British 146 Han Chinese	Not reported	GAS 20-point improvement or personal interview	Not reported	Not reported
(74)	HTR2A	Sample 1–160 Sample 2–114 White British	3 months	GAS 20-point improvement	125–600 mg	Not reported
(75)	5-HTT	268 White British	3 months	GAS 20-point improvement	Not reported	Not reported
(76)	ADRA2A ADRA1A DRD3 HTR2A HTR2C HTR3A HTRA5 5-HTT HRH1 HRH2	200 White British	Not reported	GAS “retrospective evaluation”	Not reported	Not reported
(77)	DRD3	92 Turkish	16 weeks	BPRS, SAPS and SANS 30% reduction	308.2 mg (mean)	Not reported
(78)	HTR5A	269 White British	3 months	GAS “retrospective evaluation”	Not reported	Not reported
(79)	ADRA1A ADRA2A	289 White British	3 months	GAS 20-point improvement	Not reported	Not reported
(80)	COMT 5- HTR1A	107 Italian	12 weeks	PANSS 30% reduction	229 mg (mean)	Not reported
(81)	ITIH3	143 American	6 months	BPRS 25% reduction	Not reported	Not reported
(82)	22q11.2 deletion	40 Canadian	Not reported	CGI	325 mg (mean)	Not reported
(53)	CYP2D6	34 German	10 weeks	BPRS 20% reduction	Responders: 320 mg (mean) Non-responders: 313 mg (mean)	Responders: 211ng/mL (mean) Non-responders: 269 ng /mL (mean)
(83)	Polygenic risk score	123 German	Not reported	4 level ordinal physician-rated scale of improvement	Not reported	Not reported
(84)	HTR3A HTR3B	266 White British	3 months	GAS 20-point improvement	Not reported	Not reported
(85)	GRIN2B	100 Han Chinese	8 weeks	BPRS 20% reduction	Not reported	Not reported
(86)	BDNF	93 Han Chinese	8 weeks	BPRS 20% reduction	Not reported	Not reported
(87)	APOE	95 Chinese	8 weeks	BPRS	275.5 mg (mean)	Not reported
(88)	DRD2	183 White American 49 African Americans	6 months	BPRS 20% reduction	Not reported	Not reported
(89)	DRD2	97 White American 35 African Americans	6 months	BPRS, BPOS, BNEG	Not reported	Not reported
(90)	DRD1	183 White American 49 African Americans	6 months	BPRS 20% reduction	Not reported	Not reported
(91)	DRD3	183 White American 49 African American	6 months	BPRS 20% reduction	Not reported	Not reported
(92)	GRIN1 GRIN2A GRIN2B DRD1 DRD2 DRD3	183 White American 49 African American	6 months	BPRS 20% reduction	Not reported	Not reported
(93)	DRD4 DRD5	183 White American	6 months	BPRS 20% reduction	Not reported	Not reported
(94)	DRD2	151 White American 42 African American 15 others	6 months	BPRS 20% reduction	Not reported	Not reported
(95)	NTSR1	196 White British	3 months	GAS 20 point reduction	Not reported	Not reported
(96)	5-HTT	188 White German	5 weeks	CGI, PANSS	50–800 mg	Not reported
(97)	GNB3	121 European	3 months	BPRS 30% reduction	540.91 mg (mean)	Not reported
(98)	5-HTT	116 European	3 months	BPRS 30% reduction	539.22 mg (mean)	Not reported
(45)	DRD4	74 Israeli (including Jews of European, North African and Asian origin)	16 months	Retrospective interview	365 mg (mean)	Not reported
(54)	ABCB1 ADRA1A ADRA2A ANKK1 CHRM1 CYP1A2 CYP2C19 CYP2D6 CYP3A4 CYP3A43 CYP3A5 CYP3A7 DRD1 DRD2 DRD3 DRD4 DTNBP1 GNB3 GSK3B HRH1 HTR2A HTR3A HTR6 SLC6A4 UGT1A3 UGT1A4	96 Korean	6 months	CGI score	Responders: 353.1 mg (mean) Non-responders: 312.2 mg (mean)	Responders: 662.4 ng/mL (mean) Non-responders: 627.2 ng/mL (mean)
(99)	NRXN1	163 European-American	6 months	BPRS 20% reduction	Not reported	Not reported
(100)	HTR2A	97 Chinese	8 weeks	BPRS	Not reported	Not reported
(101)	HTR2A ADRA1A ADRA2A ADRB3 GNB3	93 Taiwanese	3 months	CGI score of 1 or 2	388.2 mg (mean)	Not reported
(102)	HTR2A	70 American	10 weeks	BPRS 20% reduction	405 mg (mean)	Not reported
(103)	HTR2C	66 American	10 weeks	BPRS 20% reduction	409 mg (mean)	Not reported
(104)	DRD3	68 American	4 and 10 weeks	BPRS 20% reduction	4 week group: 497 mg (mean) 10 week group: 408 mg (mean)	Not reported
(105)	HRH1 HRH2	158 White British	3 months	GAS 20-point improvement	Not reported	Not reported
(106)	HTR2A HTR2C	144 White American 40 African American 1 Asian American	6 months	BPRS 20% reduction OR 15–20% reduction in BPRS score and a reduction of 1+ CGI category	Not reported	Not reported
(107)	HTR6	144 White American 40 African American 1 Asian American	6 months	BPRS 20% reduction OR 15–20% reduction in BPRS score and a reduction of 1+ CGI category	Not reported	Not reported
(43)	FKBP5 NR3C1 BDNF NTRK2	591 White British	3 months	GAS 20-point improvement	Not reported	Not reported
(30)	GNB3	77 White American 57 African American 11 Other American	11 weeks	BPRS	Not reported	Not reported
(44)	HTR2A	146 German	4 weeks	GAS 20 point improvement	100 mg+	Not reported
(108)	BDNF	120 European	8 weeks	PANSS 50% reduction	100–500 mg	Not reported
(109)	DRD1 DRD3 HTR2A HTR2C	13 White American 2 African American	5 weeks	BPRS	460 mg (mean)	Not reported
(49)	HTR3A	101 South Indian	12 weeks	BPRS total scores ≤35	340.84 mg (mean)	550.53 ng/mL (mean)
(110)	DRD4	29 American	20 weeks	BPRS 20% reduction AND BPRS score less than 36 or Bunney-Hamburg Global Psychosis Rating of less than 6 (mild psychosis)	”moderate” dose for first 5 weeks; ”optimized” dose for 15 weeks	Not reported
(111)	DRD4	148 German	10 weeks	GAS 20-point improvement AND BPRS 20% reduction AND BPRS score less than 36 or Bunney-Hamburg Global Psychosis rating less than 6	451.1 mg (mean)	Not reported
(112)	HTR2C	231 German	4 weeks	SADS-L	Male-−423.4 mg (mean) Female-−407.9 mg (mean)	Not reported
(113)	DRD3	32 Pakistani	6 months	BPRS 50% reduction	<600 mg	Not reported
(114)	DRD4	147 White European 42 Taiwan Chinese	3 months	GAS 20-point improvement	150–900 mg	Not reported
(115)	DRD3	183 White European	3 months	GAS 20-point improvement	150–900 mg	Not reported
(116)	HTR2A HTR2C	162 White European	3 months	GAS 20-point improvement	125–600 mg	Not reported
(117)	GPX1 MNSOD	171 White American 45 African American	6 months	BPRS 20% reduction	Not reported	Not reported
(118)	HTR3A HTR3B	114 White American 26 African American	6 months	BPRS 20% reduction	Not reported	Not reported
(119)	GFRA1 GFRA2 GFRA3 GFRA4	114 White American 26 African American	6 months	BPRS 20% reduction	Not reported	Not reported
(120)	OXT OXTR	114 White American 26 African American	6 months	BPRS 20% reduction	Not reported	Not reported
(121)	GSK3	114 White American 26 African American	6 months	BPRS 20% reduction	Not reported	Not reported
(122)	NRXN1	114 White American 26 African American	6 months	BPRS 20% reduction	Not reported	Not reported
(123)	GRIN2B	175 Europeans	6 months	BPRS 20% reduction	453 mg (mean)	Not reported
(124)	5-HTT	90 Han Chinese	8 weeks	BPRS	272 mg (mean)	Not reported
(125)	ADRA2A	97 Han Chinese	8 weeks	BPRS 20% reduction	276 mg (mean)	Not reported
(126)	TNF	99 Han Chinese	4 months	BPRS	275.5 mg (mean)	Not reported
(91)	TNF	55 Chinese	14 months	CGI score of 1 or 2	400 mg (mean)	Not reported
(50)	SLC6A3	160 Han Chinese	8 weeks	BPRS 40% reduction	300–600 mg	434 ng/mL (mean)
(127)	ABCB1 ACSM1 AGBL1 AKT1 ANK3 BDNF COMT CYP1A2 CYP2C19 CYP2C9 CYP2D6 CYP3A4 DRD2 DRD3 GRM3 HTR2C NOTCH4 PLAA RELN SHISA9 SLC1A1 SLC6A2 SLC6A3 TCF4 TNIK	240 Han Chinese	2 months	PANSS 50% reduction	122 mg (mean)	Not reported
(128)	HTR6	99 Chinese	8 weeks	BPRS 20% reduction	Not reported	Not reported
(129)	TNF-α	71 White American 25 African American	6 weeks, 3 months, 6 months	BPRS	Not reported	Not reported
(51)	DRD4	81 Han Chinese	2 months	PANSS 50% reduction	200–450 mg	712.1 ng/mL (mean)
(130)	DTNBP1	58 European American 27 African American	3 months	PANSS 20% reduction	203 mg (mean)	Not reported

*BPRS, Brief Psychiatric Rating Scale; CGI, Clinical Global Impression; GAF, Global Assessment of Functioning; PANSS, Positive and Negative Syndrome Scale; SADS-L, Schedule for Affective Disorders and Schizophrenia; SAPS, Scale for the Assessment of Positive Symptoms; SANS, Scale for the Assessment of Negative Symptoms*.

**Table 9:** Results for individual genetic variants.

	**Polymorphism**	**Study**	**Significant findings (N or Y)**	**Association with good response to clozapine (unless stated otherwise)**
22q11.2 deletion		(82)	N	–
ABCB1	rs10248420	(54)	Y	G allele
	rs10276036	(54)	N	–
	rs10280101	(54)	N	–
	rs1045642	(54)	N	–
		(127)	N	–
	rs1128503	(54)	N	–
		(127)	N	–
	rs11983225	(54)	N	–
	rs12720067	(54)	N	–
	rs1978095	(127)	N	–
	rs2032582	(54)	N	–
		(127)	Y	C allele
	rs2032583	(54)	N	–
	rs2235015	(54)	N	–
	rs3213619	(54)	N	–
	rs35023033	(54)	N	–
	rs35730308	(54)	N	–
	rs35810889	(54)	N	–
	rs3747802	(54)	Y	A allele
	rs4148739	(54)	N	–
	rs4148740	(54)	N	–
	rs72552784	(54)	N	–
	rs7787082	(54)	N	–
	rs9282564	(54)	N	–
ACSM1	rs433598	(127)	N	–
ADRA1A	Arg492Cys	(76)	N	–
		(79)	N	–
		(101)	N	–
	rs1048101	(54)	N	–
ADRA2	−1291-C/G	(76)	N	–
		(79)	N	–
		(101)	N	–
		(125)	N	–
	−261-G/A	(76)	N	–
		(79)	N	–
	rs1800038	(54)	N	–
	rs1800763	(54)	N	–
	rs521674	(54)	N	–
	rs553668	(54)	N	–
	rs602618	(54)	N	–
ADRB3	Trp64Arg	(101)	N	–
AGBL1	rs16977195	(127)	N	–
AKT1	rs2494732	(127)	N	–
	rs2494738	(127)	N	–
	rs3001371	(127)	Y	T allele
	rs3803300	(127)	N	–
ANKK1	rs10891545	(54)	N	–
	rs11604671	(54)	N	–
	rs17115439	(54)	N	–
	rs1800497	(54)	N	–
	rs4938013	(54)	N	–
ANK3	rs10761482	(127)	N	–
APOE	E4 positive or negative	(87)	N	–
BDNF	rs6265 (val66met)	(86)	N	–
		(43)	N	–
		(108)	N	–
		(127)	N	–
	rs11030076	(43)	N	–
	rs11030096	(43)	N	–
	rs1552736	(43)	N	–
CHRM1	rs2067477	(54)	N	–
COMT	rs1544325	(127)	N	–
	rs165599	(127)	N	–
	rs174696	(127)	N	–
	rs174697	(127)	N	–
	rs174699	(127)	N	T allele
	rs4646312	(127)	N	–
	rs4646316	(127)	Y	–
	rs4680 (Val158Met)	(80)	N	–
		(127)	N	–
	rs4818	(127)	N	–
	rs5993883	(127)	N	–
	rs6269	(127)	N	–
	rs737865	(127)	N	–
CYP1A2	rs762551	(54)	N	–
		(127)	N	–
	rs12720461	(54)	N	–
	rs2069521	(54)	N	–
	rs2069522	(54)	N	–
	rs2069526	(54)	N	–
	rs2470890	(54)	N	–
	rs55889066	(54)	N	–
	rs72547516	(54)	N	–
CYP2C19	rs11188072	(54)	N	–
	rs11568732	(54)	N	–
	rs12248560	(54)	N	–
	rs17884712	(54)	N	–
	rs2104161	(127)	N	–
	rs41291556	(54)	N	–
	rs4244285	(54)	N	–
		(127)	N	–
	rs4986893	(54)	N	–
		(127)	N	–
	rs4986894	(54)	N	–
	rs56337013	(54)	N	–
		(127)	N	–
CYP2C9	rs1057910	(127)	N	–
	rs1934969	(127)	N	–
CYP2D6	Unspecified	(72)	N	–
		(53)	N	–
	rs1065852	(54)	N	–
		(127)	N	–
	rs1135840	(54)	N	–
		(127)	N	–
	rs16947	(54)	N	–
		(127)	N	–
	rs28371720	(54)	N	–
	rs28371725	(54)	N	–
	rs3892097	(54)	N	–
		(127)	N	–
	rs4986774	(54)	N	–
	rs5030655	(54)	N	–
	rs59421388	(54)	N	–
	rs61736512	(54)	N	–
CYP3A4	rs2242480	(127)	Y	C allele
	rs2246709	(54)	N	–
	rs2740574	(54)	N	–
	rs28371759	(54)	N	–
		(127)	N	–
	rs4986907	(54)	N	–
	rs4986909	(54)	N	–
	rs4986910	(54)	N	–
	rs4986913	(54)	N	–
	rs4987161	(54)	N	–
CYP3A43	rs17342647	(54)	N	–
	rs61469810	(54)	N	–
	rs680055	(54)	N	–
CYP3A5	rs10264272	(54)	N	–
	rs776746	(54)	N	–
CYP3A7	rs2257401	(54)	N	–
DTNBP1	rs1018381	(130)	N	–
	rs2619538	(54)	N	–
	rs2619539	(54)	N	–
		(130)	N	–
	rs3213207	(54)	N	–
	rs742105	(54)	N	–
		(130)	Y	T allele
	rs742106	(130)	N	–
	rs760761	(130)	N	–
	rs909706	(54)	N	–
		(130)	N	–
DRD1	rs265976	(90)	Ya	AC genotype—non responders
	rs265981	(90)	N	–
	rs4532 (−48 AG)	(90)	N	–
		(109)	Y	“2/2 genotype”
	rs5328	(54)	N	–
	rs686	(90)	N	–
DRD2	−141 Ins/Del C	(55)	N	–
		(88)^*^	N	–
	rs1076560	(54)	N	–
	rs1076562	(127)	N	–
	rs1079598 (Taq1B C/T)	(88)^*^	Ya	T allele
	rs1079727	(127)	N	–
	rs1125394 A/G	(88)^*^	Ya	A allele
		(127)	N	–
	rs12364283	(54)	N	–
	rs1799978	(88)^*^	N	–
		(54)	N	–
		(127)	N	–
	rs1800497	(88)^*^	Ya	C allele
		(127)	N	–
	rs1800498 (Taq1D C/T)	(88)^*^	N	–
	rs1801028 (Ser311Cys)	(54)	N	–
	rs2075652	(127)	N	–
	rs2242591 A/G	(88)^*^	N	–
	rs2242592 C/T	(88)^*^	N	–
	rs2242593 A/G	(88)^*^	N	–
	rs2283265	(54)	N	–
		(127)	N	–
	rs2514218 A/G	(94)	Yw	A allele
	rs4648317 C/T	(88)^*^	N	–
	rs4648318	(127)	N	–
	rs6275 (NcoI C/T)	(88)^*^	N	–
		(54)	N	–
		(104)	N	–
		(115)	Y	Gly 9 allele
		(127)	N	–
		(77)	N	–
	rs6277 (C957T)	(88)^*^	N	–
		(91)	N	–
		(54)	N	–
		(127)	N	–
	rs7103679	(127)	N	–
	rs7131056	(127)	N	–
DRD3	rs1394016	(91)	N	–
	rs167770	(91)	N	–
	rs167771	(54)	N	–
		(127)	Y	G allele
	rs2087017	(91)	N	–
	rs2134655	(91)	Yw	A allele
	rs2399504	(91)	N	–
	rs324036	(127)	N	–
	rs6280 (Ser-9-Gly)	(76)	N	–
		(77)	N	–
		(91)	N	–
		(54)	N	–
		(104)	N	–
		(113)	Y	Gly 9 allele
		(115)	Y	Gly 9 allele
		(109)	N	–
		(127)	N	–
	rs6762200	(91)	N	–
	rs7611535	(91)	N	–
	rs905568	(91)	N	–
	rs963468	(127)	N	–
DRD4	12 bp repeat	(45)	N	–
		(111)	N	–
	13 bp repeat	(111)	N	–
	48 bp repeat	(93)	N	–
		(45)	N	–
		(110)	N	–
		(111)	N	–
		(114)	N	–
		(51)	Y	5 allele—non-responders
	120 bp repeat	(93)	N	–
	G(n) repeat	(93)	N	–
	Gly11Arg	(111)	N	–
	rs11246226	(93)	N	–
		(54)	N	–
	rs3758653	(93)	N	–
		(54)	N	–
	rs916457	(54)	N	–
	rs936465	(93)	N	–
DRD5	CA/CT/GT dinucleotide microsatellite repeat	(93)	N	–
	rs10001006	(93)	N	–
	rs10033951	(93)	N	–
	rs1967551	(93)	N	–
	rs6283	(93)	N	–
FKBP5	rs1360780	(43)	Y	C allele
	rs17542466	(43)	N	–
	rs2766533	(43)	N	–
	rs3777747	(43)	N	–
GFRA1	rs1078080	(118)	N	–
	rs10749189	(118)	N	–
	rs10787627	(118)	N	–
	rs10885877	(118)	N	–
	rs10885888	(118)	N	–
	rs11197557	(118)	N	–
	rs11197567	(118)	N	–
	rs11197612	(118)	N	–
	rs11598215	(118)	N	–
	rs11812459	(118)	N	–
	rs12413585	(118)	N	–
	rs12775655	(118)	N	–
	rs12776813	(118)	N	–
	rs17094340	(118)	N	–
	rs2694783	(118)	N	–
	rs2694801	(118)	N	–
	rs3781514	(118)	N	–
	rs3781539	(118)	N	–
	rs3824840	(118)	N	–
	rs4751956	(118)	N	–
	rs7085306	(118)	N	–
	rs730357	(118)	N	–
	rs7903297	(118)	N	–
	rs7920934	(118)	N	–
	rs9787429	(118)	N	–
GFRA2	rs15881	(118)	N	–
	rs10088105	(118)	N	–
	rs10283397	(118)	N	–
	rs1128397	(118)	N	–
	rs11993990	(118)	N	–
	rs13250096	(118)	N	–
	rs4078157	(118)	N	–
	rs4237073	(118)	N	–
	rs4567027	(118)	N	–
	rs4567028	(118)	N	–
	rs4739217	(118)	N	–
	rs4739285	(118)	N	–
	rs4739286	(118)	N	–
	rs6587002	(118)	N	–
	rs6988470	(118)	N	–
	rs7014143	(118)	N	–
	rs7813735	(118)	N	–
GFRA3	rs10036665	(118)	N	–
	rs10952	(118)	N	–
	rs11242417	(118)	N	–
	rs7726580	(118)	N	–
GFRA4	rs6084432	(118)	N	–
	rs633924	(118)	N	–
GNB3	rs1129649	(54)	N	–
	rs3759348	(54)	N	–
	rs5439	(54)	N	–
	rs5440	(54)	N	–
	rs5441	(54)	N	–
	rs5442	(54)	N	–
	rs5443 (C825T)	(97)	Y	C allele
		(54)	N	–
		(101)	N	–
		(30)	Yw	C/C genotype
	rs5446	(54)	N	–
GPX1	rs1050450 (Pro200Leu)	(117)	N	–
GRIN1	rs11146020 (G1001C)	(92)	N	–
GRIN2A	GT dinucloedtide repeat microsatellite polymorphism in promoter region	(92)	N	–
GRIN2B	rs10193895 (G-200T)	(92)	N	–
	rs1072388	(123)	N	–
	rs12826365	(123)	N	–
	rs1806191	(123)	N	–
	rs1806201 (C2664T)	(85)	N	–
		(123)	N	–
	rs2284411	(123)	N	–
	rs3764030	(123)	N	–
	rs890	(123)	N	–
GRM3	rs274622	(127)	N	–
	rs724226	(127)	N	–
GSK3B	rs11919783	(121)	N	–
	rs11923196	(121)	N	–
	rs13319151	(121)	N	–
	rs13321783	(54)	N	–
	rs2319398	(54)	N	–
	rs334558	(54)	N	–
	rs3755557	(121)	N	–
	rs3755557	(121)	N	–
	rs4072520	(121)	N	–
	rs4491944	(121)	N	–
	rs4688043	(121)	N	–
	rs6438552	(121)	N	–
	rs6772172	(121)	N	–
	rs6779828	(121)	N	–
	rs6805251	(121)	N	–
	rs6808874	(54)	N	–
	rs7624540	(121)	N	–
	rs9846422	(121)	N	–
	rs9846422	(121)	N	–
	rs9878473	(121)	N	–
HRH1	−17-C/T	(105)	N	–
	Leu449Ser	(76)	N	–
	−974-C/A	(105)	N	–
	−1023-A/G	(105)	N	–
	−1536-G/C	(105)	N	–
	rs12490160	(54)	N	–
	rs13064530	(54)	N	–
	rs6778270	(54)	N	–
HRH2	−1010- G/A	(76)	N	–
	−294-A/G	(105)	N	–
	−592-A/G	(105)	N	–
	−1018-G/A	(105)	N	–
	−1077-G/A	(105)	N	–
HTR1A	C->T 47	(107)	N	–
	rs6295 (−1019 C/G)	(80)	N	–
HTR2A	his452tyr	(73)	Y	His allele
		(74)	Y	His allele
		(76)	Y	His allele
		(102)	N	–
		(106)	Y	His allele
		(44)	N	–
	Thr25Asp	(76)	N	–
		(44)	N	–
	516-C/T	(76)	N	–
	rs6311 (G-1438A)	(106)	N	–
		(74)sample 1	Y	GG genotype—non-responders
		(74) sample 2	N	–
		(76)	Y	Not reported
		(54)	N	–
	rs6313 (T102C)	(71)	Y	T102 allele
		(76)	Y	T102 allele
		(54)	N	–
		(100)	N	–
		(101)	N	–
		(102)	N	–
		(106)	N	–
		(44)	N	–
		(109)	N	–
		(116)	Y	T102 allele
	rs7997012	(54)	N	–
	rs9316233	(54)	N	–
HTR2C	Cys23ser	(116)	Y	Ser allele
		(106)	N	–
		(76)	N	–
		(109)	N	–
		(112)	N	–
		(103)	N	–
	−330–GT/ 244–CT repeat	(76)	Y	Not reported
	rs1023574	(127)	N	–
	rs1414334	(127)	N	–
	rs2192371	(127)	N	–
	rs3813929	(127)	Y	C allele
	rs498177	(127)	Y	G allele
	rs518147	(127)	N	–
	rs5988072	(127)	N	–
	rs9698290	(127)	N	–
HTR3A	rs1062613 (178-C/T)	(76)	N	–
		(84)	N	–
		(54)	N	–
		(49)	Y	T allele
		(118)	Y	C allele
	rs1150226	(118)	N	–
	rs1176713	(54)	N	–
		(118)	N	–
	rs2276302 (1596-A/G)	(76)	N	–
		(84)	N	–
		(49)	Y	G allele
		(118)	N	–
HTR3B	a CA repeat polymorphism	(84)	N	–
	rs1176744	(118)	N	–
	rs2276307	(118)	N	–
	rs3758987	(118)	N	–
	rs3782025	(118)	N	–
HTR5	−19G/C	(76)	N	–
		(78)	N	–
	12A/T	(76)	N	–
		(78)	N	–
HTR6	T->C 267	(107)	N	–
		(128)	Y	TT genotype
	rs1805054	(54)	N	–
HTR7	pro279leu	(107)	N	–
5HTT	VNTR	(76)	N	–
		(75)	N	–
	VNTR Stin2	(96)	N	–
	Ins/Del 44 bp	(96)	N	–
		(98)	N	–
	484 vs. 528 bp	(124)	N	–
	rs6352	(54)	N	–
	rs2020934	(54)	N	–
	HTTLPR repeat	(76)	Y	Not reported
		(98)	Y	Long allele
		(75)	N	–
ITIH4	rs2535629	(81)	Y	A allele^a^^a^
MNSOD	rs4880 (Ala16Val)	(117)	N	–
NOTCH4	rs3131296	(127)	N	–
NR3C1	rs1837262	(43)	N	–
	rs2963156	(43)	N	–
	rs4634384	(43)	N	–
	rs4912910	(43)	N	–
NRXN1	rs1045881 C/T	(99)	Y	C allele
	rs10490162	(122)	N	–
	rs12467557	(122)	N	–
	rs1400882	(122)	N	–
	rs17041112	(122)	N	–
NTRK2	rs10465180	(43)	Y	T allele
	rs1619120	(43)	N	–
	rs1778929	(43)	Y	C allele
	rs4388524	(43)	N	–
NTRS1	3020-T/C	(95)	N	–
	VNTR in 3′-flanking region	(95)	N	–
OXT	rs2740204	(120)	Y	G allele
	rs2740210	(120)	N	–
	rs2770378	(120)	N	–
	rs3761248	(120)	N	–
	rs4813625	(120)	N	–
	rs877172	(120)	N	–
OXTR	rs1042778	(120)	N	–
	rs11131149	(120)	N	–
	rs11706648	(120)	N	–
	rs2268492	(120)	N	–
	rs2268496	(120)	N	–
	rs237884	(120)	N	–
	rs237885	(120)	N	–
	rs237887	(120)	N	–
	rs237889	(120)	N	–
	rs237894	(120)	N	–
	rs237897	(120)	N	–
	rs237899	(120)	N	–
	rs4686301	(120)	N	–
	rs9840864	(120)	N	–
PLAA	rs7045881	(127)	N	–
RELN	rs7341475	(127)	N	–
SHISA9	rs7192086	(127)	N	–
SLC1A1	rs2228622	(127)	N	–
SLC6A2	rs5569	(127)	Y	G allele
	rs2242446	(127)	N	–
SLC6A3	30-bp VNTR in intron 8	(50)	N	–
	40-bp VNTR in the 3′-region	(50)	N	–
	rs2652511	(50)	N	–
	T-844C	(127)	N	–
	rs27072	(50)	N	–
	rs2963238 (A1491C)	(50)	N	–
		(127)		–
	rs2975226 (T-71A)	(50)	Y	T allele
TCF4	rs9960767	(127)	Y	A allele
	rs17594526	(127)	N	–
TNF	−308G/A	(126)	N	–
		(91)	N	–
		(129)	Y	A allele
TNIK	rs2088885	(127)	Y	A allele
UGT1A3	rs10929302	(54)	N	–
	rs28898605	(54)	N	–
	rs28934877	(54)	N	–
	rs3732218	(54)	N	–
	rs3732220	(54)	N	–
	rs3806591	(54)	N	–
	rs3806595	(54)	N	–
	rs4124874	(54)	N	–
	rs4148323	(54)	N	–
	rs869283	(54)	N	–
	rs887829	(54)	N	–

* *Hwang et al. (89) used a subset of the Hwang et al. (88) sample so results for the same polymorphisms from the 2006 paper have not been reported*,

a *Result only in European samples*.

**Table 10:** Significant findings for haplotypes.

**Gene**	**Alleles**	**Study**	**Association**
DRD1	rs265981-T rs4532-G rs686-A	(90)	Response^a^
	rs265981-T rs4532-G rs686-G	(90)	Response^b^
DRD2	rs1125394-A rs1079598 (TaqIB)-T Taq1A-C	(88)	Response^b^
	rs1079598 (TaqIB)-T Taq1D-T NcoI-C	(88)	Response^a^
	Taq1D-T NcoI-C C957T-T	(88)	Response^a^
	−141 Ins rs4648317-C rs1125394-A	(88)	Response^b^
	rs4648317-C rs1125394-A rs1079598 (TaqIB) - T	(88)	Response^b^
	rs1125394-A rs1079598 (TaqI B) - T Taq1D-T	(88)	Response^b^
	rs2242592-C rs2242593-A Taq1A-C	(88)	Response^b^
	rs1125394-A rs1079598 (TaqIB)-T	(89)	Response^b^
	rs4648317-C rs1125394-A rs1079598 (TaqIB)-T	(89)	Response^b^
	rs1125394-A rs1079598 (TaqIB) - T rs1800498 (TaqID) - C	(89)	Response^b^
DRD3	rs6280-A rs167770-C rs2134655-G	(91)	Non-response^a^
	rs6280-A rs167770-C	(91)	Non-response^a^
	rs6280-A rs167770-T	(91)	Response^a^
	rs905568-C rs2399504-A rs7611535-A	(91)	Response^a^
	rs7611535-G rs6762200-G rs1394016-C	(91)	Response^b^
	rs6762200-A rs1394016-T rs6280-G	(91)	Response^b^
	rs6762200-G rs1394016-C rs6280-G	(91)	Response^b^
	rs1394016-C rs6280-G rs167770-C	(91)	Response^a^
	rs7611535-G rs6762200-G rs1394016-T	(91)	Non-response^a^
	rs7611535-A rs6762200-A rs1394016-C	(91)	Non-response^b^
	rs167770-C rs2134655-G	(91)	Non-response^a^
	rs7611535-A rs6762200-A	(91)	Non-response^b^
	rs2399504-G rs7611535-G	(91)	Response^b^
	rs6762200-G rs1394016-T rs6280-G	(91)	Response^b^
FKBP5	rs3777747-A rs1360780-T rs17542466-A rs2766533-G	(43)	Non-response
GFRA2	rs1128397-T rs13250096-G rs4567028-G	(119)	Response
HTR3A	rs2276302-A rs1062613-C rs1150226-C	(118)	Response
NTRK2	rs1619120-G rs1778929-T rs10465180-C	(43)	Non-response
	rs1619120-G rs1778929-C rs10465180-T	(43)	Response

a *Result only in White participants*,

b *Result only in African-American participants*.

**Table 11:** Significant gene-gene interaction results.

**Genes**	**Polymorphisms**	**Study**
DRD1; DRD3	rs686; Ser9Gly	(92)^a^
	rs4532; rs1394016	(92)^a^
DRD2; DRD3	Taq1b; rs2134655	(92)^a^
	C975T; Ser9Gly	(92)^b^
DRD1; GRIN2A	rs265976; GTrepeat	(92)^b^
GFRA1; GFRA2; GFRA3	rs10885888; rs4237073; rs7726580	(119)
HTR2A; HTR2A; HTR2C; HTR2C; SLC6A4; HRH1	T102C; His452Tyr; G-330T / C-244T repeat; Cys23Ser; HTTLPR; G-1018A	(76)
HTR2A; ADRA1A; ADRA2A; ADRB3; GNB3; plus clinical information in artificial neural network	T102C; Arg347Cys; −1291C>G; Trp64Arg; 825C>T	(101)

a *Result only in White participants*,

b *Result only in African-American participants*.

Of the other genetic studies, seven reported significant associations between genetic haplotypes of DRD1, DRD2, DRD3, FKBP5, GFRA2, HTR3A, and NTRK2 (43, 88–91, 119) (see Table 10) and clozapine response, but none of these have been replicated. Two unreplicated studies also reported significant associations between gene-gene interactions of DRD1 and DRD2, DRD2 and DRD3, DRD1 and GRIN2A, and GFRA1, GFRA2, and GFRA3 (92, 119) and clozapine response (see Table 11).

Two studies reported the predictive validity of multivariate genetic models. One study investigated a logistic regression analysis with a combination of six polymorphisms (T102C and His452Tyr of HTR2A gene, G-330T/ C-244T repeat and Cys23Ser of HTR2C gene, HTTLPR of SLC6A4 gene, G-1018A of HRH1) which was able to predict clozapine response with the retrospective positive predictive value of 76.7%, negative predictive value of 82%, a sensitivity of 95% and specificity of 38% (76). A more recent study used an artificial neural network analysis to combine five genetic polymorphisms (T102C of the HTR2A gene, Arg347Cys of the ADRA1A gene, −1291 C>G of the ADRA2A gene, Trp64Arg of the ADRB3 gene, and 825 C>T of the GNB3 gene), which were insignificant individually, with clinical predictor variables (gender, age, height, baseline body weight, baseline body mass index) (101). This approach was able to retrospectively identify all clozapine responders and 76.5% clozapine non-responders.

However, our search mainly returned studies that have employed candidate gene approaches to investigation of clozapine response. Overall, these studies have investigated associations with clozapine response for a total of 379 different gene variants, 362 of which relate to single nucleotide polymorphisms (SNPs). For these studies, we limit comment to significant findings with at least one replication. Of the 379 different gene variants investigated, significant findings have been reported for 40 variants, 8 of which have been replicated. 28 variants have replicated null results with no significant findings, including the rs6275 and rs6277 polymorphisms of DRD2 (54, 88, 127) and the val66met polymorphism in BDNF (43, 86, 108, 127). The details for all genetic studies, including those with non-significant or non-replicated findings, are provided in Table 9.

#### Dopaminergic genes

The DRD3 gene, encoding the D_3_ dopamine receptor, has been investigated in nine studies, all of which have investigated the Ser9Gly polymorphism of rs6280. While two initial studies independently reported that the Gly allele was associated with a good response to clozapine (113, 115), all seven subsequent studies found non-significant results (54, 76, 77, 91, 104, 109, 127), including the two studies with the largest sample size (76, 91).

#### Serotonergic genes

The HTR2A gene, encoding the 5-HT_2A_ receptor at which clozapine has high affinity, has been investigated in 12 studies. The His allele of His452Tyr has been associated with good response to clozapine in four studies conducted by two research groups (73, 74, 76, 106), although two studies did not detect this association (44, 102). Within the same gene, the T allele of the T102C polymorphism has been associated with good response to clozapine in three studies by the same research group (71, 76, 116), although seven studies by other groups have failed to replicate these findings (44, 54, 100–102, 106, 109). The G-1438A SNP also significantly predicted clozapine response in two studies by the same group (74, 76) but these results were not replicated in a second sample analyzed by the same research group (74) or in separate samples from two independent research groups (54, 106).

The HTR3A gene has been investigated in five studies (49, 54, 76, 84, 118); the only SNP which has been reported more than once, across all five studies, is rs1062613, with one study finding that good response to clozapine was associated with the T allele (49), another finding that good clozapine response was associated with the C allele (118) and the other three studies reporting no association.

The 5HTT (or SLC6A4) gene, encoding the serotonin transporter, has been investigated in six studies by five independent groups (54, 75, 76, 96, 98, 124), with the only independently replicated finding for an association of the HTTLPR polymorphism at rs25531 with clozapine response; Kohlrausch et al. (98) found an association between good response and the long allele, but Arranz et al. (76) do not report the direction of effect.

#### Other gene variants

An association between the C allele of the C825T polymorphism in the gene encoding G-protein subunit-beta 3 (GNB3) and a good response to clozapine has been reported in two studies performed by independent research groups (30, 97), though two separate studies by two other research groups have found no association (54, 101).

## Discussion

Since 1992, ninety-eight published studies have tested biological predictors of symptomatic response to clozapine. While this highlights the potential clinical importance of identifying good clozapine responders in advance of starting treatment, these 25 years of research have failed to produce biomarkers with sufficient accuracy for clinical decision making. The most consistent findings are that a good response to clozapine is associated with greater structural integrity and activity in prefrontal cortical areas, possibly reflecting less severe brain pathophysiology than in poor responders, and a lower ratio of the dopamine metabolite HVA to the serotonin metabolite 5-HIAA in CSF before clozapine initiation, reflecting higher serotonergic compared to dopaminergic turnover. However, there have been relatively few studies investigating these biomarkers prospectively and further replication is required.

Regarding prefrontal cortical areas, prospective studies have found consistent evidence that higher prefrontal cortical volumes before clozapine initiation are directly associated with a greater degree of symptomatic response to clozapine (31, 36, 37), with some suggestion of specificity to improvements in negative symptom severity (31, 37). Studies examining perfusion or metabolism have similarly associated higher levels of prefrontal activity with a higher degree of symptomatic response (32, 37, 41). These results are consistent with the majority, but not all (132) of cross-sectional studies finding that clozapine responders have higher prefrontal cortical volumes than non-responders (25, 27, 28). In addition, some evidence indicates that integrity/activity of the thalamus may also be important in predicting clozapine response (32, 37, 41). Importantly, the jack-knifed classification of Rodriguez et al. (41) using DLPFC and thalamic activity correctly identified 78.9% cases according to clozapine response, and the effect size of the difference in prefrontal sulcal widening score between clozapine responders and non-responders reported by Konicki et al. (36) can be calculated as a large effect size of *d* = 3.8.

It is unclear whether prefrontal structural integrity or activity may be predictive of clozapine response specifically, or whether prefrontal integrity is non-specifically prognostic of outcome. Findings relating prefrontal volume to symptom outcomes in non-clozapine treated patients are mixed (37, 133–135), with the largest study finding no relationships between gray matter volume at illness onset and outcome 2 years later (135). Some studies indicate that clozapine has greater ability to modulate prefrontal activity than other antipsychotic compounds (29, 109, 136–138), but we are not aware of any studies that have specifically compared the ability of prefrontal cortical variables to predict response to clozapine vs. other antipsychotics. Determination of treatment specificity would be important for clinical decision-making around clozapine initiation.

The other most replicated finding is that the ratio of the dopamine to serotonin metabolites HVA:5-HIAA in CSF at baseline predicted clozapine response (47, 48, 64). Where available, the effect sizes calculated for these studies are large [*d* = 0.8 (47) and 1.2 (48)]. CSF HVA and 5-HIAA respectively reflect brain dopaminergic and serotonergic turnover, with some evidence that lumbar CSF HVA is primarily from the striatum (139) and 5-HIAA from the frontal cortex (47). These findings in the absence of predictive value of CSF HVA or 5-HIAA alone suggest that the dopamine-serotonin balance is predictive of clozapine response. One report that CSF HVA/5-HIAA ratio was not predictive of response to olanzapine (140) may be suggestive of clozapine specificity, although further confirmation is needed.

In terms of genetic predictors of clozapine response, our results highlight the overall inability of candidate gene approaches to reproducibly predict clozapine response. Of the 379 polymorphisms investigated in relation to clozapine response, replication by two or more independent research groups is only available for the DRD3 Ser9Gly (113, 115), HTR2A His452Tyr (73, 74, 76, 106), 5HTT rs25531 (76, 98), and C825T GNB3 (30, 97) polymorphisms. Furthermore, findings of no association with clozapine response were also reported for DRD3 (76, 77, 91, 104, 109), HTR2A His452Tyr (44, 102), C825T GNB3 (54) and no findings were replicated by more than two independent groups. However, as is the case for schizophrenia, clozapine response is unlikely to be dictated by a single gene variant, and more likely reflects additive or interacting effects at multiple genetic loci. One study investigating a combination of six polymorphisms predicted clozapine response with the retrospective positive predictive value of 76.7% and a sensitivity of 95% (76) on which basis a pharmacogenetic test was developed, although it is no longer available. Similarly, using an artificial neural network to combine five polymorphisms with clinical data retrospectively identified all clozapine responders and 76.5% of non-responders (101).

Since many of these studies were done, technology has advanced to genome-wide association studies (GWAS), which take a hypothesis-free approach but require very large samples. GWAS is being applied to identify polymorphisms contributing to response to non-clozapine antipsychotics (141) and may be applied to clozapine in the future. This approach is encouraged by reports that polygenic risk scores for schizophrenia may associate with the degree of clozapine response (83). However, genome-wide approaches specifically comparing good vs. poor responders to clozapine are required because many of the candidate gene studies identified by our review investigated polymorphisms previously associated with non-clozapine antipsychotic response with minimal success or without replication [e.g., NRXN1: (122); ABCB1: (54, 127)], indicating that clozapine research would benefit from approaches able to identify novel genetic associations. Another avenue to explore is epigenetic variation, in the form of chemical modifications associated with differing gene expression such as DNA or histone methylation, which may play a role in clozapine response above and beyond genetic variation; evidence indicates both that variation in these modifications is associated with schizophrenia (142) and that clozapine induces changes in these modifications (143).

Our review also highlights several methodological considerations for future studies examining predictive biomarkers of clozapine response. First, there are overall relatively few studies that have prospectively examined non-genetic biological predictors of clozapine response despite their potential clinical importance. This likely reflects several practical factors. In our own experience, patients who are about to start clozapine can be difficult to recruit to research involving neuroimaging or invasive procedures, because they are often very unwell and may lack capacity to consent. Additionally, research participation needs to be approached and timed carefully around clinical conversations regarding clozapine initiation. This may partly explain the relatively few studies overall, and small sample sizes in some studies.

Secondly, although a response to clozapine will require adequate dosing, only nine of the ninety-eight studies included in our review reported clozapine plasma concentrations. Without this information it is not possible to determine the extent to which poor response may reflect sub-therapeutic plasma clozapine concentrations rather than clozapine inefficacy. There was also significant variability in criteria used to determine clozapine response/non-response as well as variability in clozapine treatment duration. Clinical trials indicate that the majority of patients who will respond to clozapine will do so in the first 6 weeks of treatment, which is associated with ~30% response (57, 144). By 12 weeks of clozapine treatment, a response is seen in 40–50% of patients (145, 146). Therefore, studies of less than 12 weeks duration may have been too short to establish clozapine response or non-response. To address some of this inconsistency, the Treatment Response and Resistance in Psychosis (TRRIP) Working Group have recently provided consensus guidelines for determining and reporting adequate treatment and treatment response (11); this includes a recommendation that clozapine therapy be maintained for a minimum of 3 months after therapeutic plasma levels are reached before determining response.

As with other biomarker research, technical constraints, and cost may impede the translation of some markers to clinical practice. Broadly, blood-based biomarkers may be more readily implemented than biomarkers requiring advanced neuroimaging techniques, lumbar puncture or specialized analysis. However, this should be balanced against the high economic costs of treatment resistant schizophrenia. Models based on clinical or demographic factors may be easier to implement. However, as for biological markers, previous reviews of clinical predictors of clozapine response have failed to identify any with “adequate reproducibility, sensitivity and specificity for clozapine,” instead suggesting that a combination of factors may be most fruitful (24). Another broader challenge is the lack of established biological underpinnings for schizophrenia and the subsequent heterogeneity in patients, which may obscure identification of biological predictors. Research indicates potential categorical differences between patients with treatment-responsive and treatment-resistant schizophrenia (12), as well as potential sub-groups within treatment-resistant patients (21), with further sub-groups likely. Such differences may contribute to the lack of reproducible research findings, and future research could explore whether predictors of outcome are specific to sub-groups within the schizophrenia diagnosis.

In conclusion, this review supports the notion that biological measures might be useful in predicting response to clozapine, and that higher prefrontal structural integrity and activity and lower ratios of HVA/5-HIAA in CSF may be associated with a better response. Future research should confirm these findings, investigate treatment-specificity, and apply genome-wide approaches. If these approaches are to aid clinical decision making, future studies will also need to address the accuracy of prediction at the individual patient level, which may be facilitated by statistical models combining neuroimaging, CSF-based, blood-based, genetic, clinical, or demographic measures.

## Author contributions

RS and AE designed the study and protocol. RS and AG conducted the systematic review. RS, AG, and AE jointly wrote the first draft of the manuscript. GM, K-VS, and JM provided additional intellectual contributions, and all authors contributed to and approved the final manuscript.

### Conflict of interest statement

The authors declare that the research was conducted in the absence of any commercial or financial relationships that could be construed as a potential conflict of interest.

## Footnotes

^1^This was clarified directly with the corresponding author for this paper due to discrepancies between the text and figure in the paper.

## Abbreviations

ACTH: adrenocorticotropic hormone
BPRS: brief psychiatric rating Scale
CGI: clinical global impression
CSF: cerebrospinal fluid
CT: computerized tomography
DLPFC: dorsolateral prefrontal cortex
ECG: electrocardiogram
EEG: electroencephalogram
GWAS: genome-wide association studies
HLA: human leukocyte antigen
5-HIAA: ty5-hydroxyindoleacetic acid
HVA: homovanillic acid
MAO-B: monoamine oxidase B
MCPP: m-chlorophenylpiperazine
MHPG: 3-methoxy-4-hydroxyphenylglycol
MRI: magnetic resonance imaging
MRS: magnetic resonance spectroscopy
NAA: n-acetyl aspartate
NMDA: N-methyl-D-aspartate
PANSS: positive and negative syndrome scale
PET: positron emission tomography
SANS: scale for the assessment of negative symptoms
SAPS: scale for the assessment of positive Symptoms
SNPs: single nucleotide polymorphisms
SPECT: single photon emission computerized tomography
TRS: treatment resistant schizophrenia.
